# Older adults preserve accuracy but not precision in explicit and implicit rhythmic timing

**DOI:** 10.1371/journal.pone.0240863

**Published:** 2020-10-19

**Authors:** Elisa M. Gallego Hiroyasu, Yuko Yotsumoto

**Affiliations:** Department of Life Sciences, The University of Tokyo, Tokyo, Japan; Waseda University, JAPAN

## Abstract

Aging brings with it several forms of neurophysiological and cognitive deterioration, but whether a decline in temporal processing is part of the aging process is unclear. The current study investigated whether this timing deficit has a cause independent of those of memory and attention using rhythmic stimuli that reduce the demand for these higher cognitive functions. In Study 1, participants took part in two rhythmic timing tasks: explicit and implicit. Participants had to distinguish regular from irregular sequences while processing temporal information explicitly or implicitly. Results showed that while the accuracy in the implicit timing task was preserved, older adults had more noise in their performance in the explicit and implicit tasks. In Study 2, participants took part in a dual-implicit task to explore whether the performance of temporal tasks differed with increasing task difficulty. We found that increasing task difficulty magnifies age-related differences.

## Introduction

Often our behavior is dependent on the accuracy with which we can process temporal information, be it consciously or unconsciously. In timing research, these have been termed as explicit and implicit timing, respectively. Whereas awareness of temporal processing is key in explicit timing, participants may not be consciously attending to time in implicit tasks. Hence, a growing number of studies have examined dissociations between explicit and implicit processing.

Dissociations between explicit and implicit timing have been shown at both the neurological and behavioral levels. At the neurological level, Coull and Nobre [1] and Weiner, Turkeltaub, and Coslett [2] suggested that unlike implicit timing, explicit timing calls upon structures such as the basal ganglia. Of the many brain areas known to be involved in the processing of time [3–5], the basal ganglia are considered the central hypothetical internal clock [3, 6]. Similarly, Mioni and colleagues [7] recently showed that while patients with Parkinson’s disease (PD), who suffer degeneration in the dopaminergic system involving the basal ganglia, perceived durations to be shorter than they actually were in the explicit task, they had intact temporal cognition in the implicit task.

Behavioral results with children, as well as beat-deaf participants, have also supported the idea that explicit and implicit timing recruit different mechanisms. Droit-Volet and Coull [8] showed that developmental trajectories were different for explicit and implicit timing; while children of five years of age performed equally well as adults in the implicit timing task, they were more variable in the explicit timing task. Moreover, a study showed that participants who presented difficulties perceiving the beats, were able to implicitly synchronize to the beat [9]. This was most likely due to the use of intact networks implicated in implicit processing of temporal information. Explicit processing and implicit processing of time, therefore, seem to recruit different neural structures and mechanisms, manifesting as differences between these two methods whereby we process time.

These studies suggest that the underdevelopment or decline in specific brain structures involved in the processing of explicit timing may contribute to dysfunctions in temporal processing. However, there are other factors that may contribute to the distorted representation of time, such as the motoric components of the task required by tapping tasks [10] and the cognitive functions of attention and memory [11, 12]. Understanding how the brain processes temporal information free from other general cognitive functions is challenging due to difficulties in dissociating temporal cognition from attention and memory [13]. Hence, it remains unclear whether the general alteration in temporal processing in the explicit task, especially in the developmental process, is a result of neurological or cognitive differences involved in the tasks.

Possible recruitment of attentional and memory demands may lead to differences in the performance of temporal tasks in older adults. It is known that older adults have reduced ability in attention. Henry, Herrmann, Kunke, and Obleser [14] found that older adults’ attention decreases in the first three seconds compared to sustained attention in younger adults. Moreover, depending on the difficulty of the task, the addition of a memory task may require competition for limited attentional resources, worsening the performance of older adults in timing tasks [15]. Thus, even with temporal tasks, the choice of task used to study temporal perception is crucial, because several tasks differ in their cognitive demands [16].

It may be for the above reasons that studies focusing on the temporal cognition of older adults have shown mixed results. The general notion under the Pacemaker-Accumulator Model [17] is that the pacemaker of the theoretical internal clock of older adults beats pulses more slowly than in younger adults [18–20]. Contrariwise, other studies have shown that there may in fact be no age-related differences in the speed of the internal clock [21, 22] or that the clocks of older adults beat at a faster rate than for their younger counterparts [23, 24]. Therefore, the way their performance varies by task challenges the concept that aging affects temporal cognition due to age-related changes in the structural changes in the hypothetical internal clock.

In this paper, we paid particular attention to the type of task given to older adults. As discussed, there are two primary components that may influence the distortions of temporal processing in older adults: the amount of attentional resources devoted to the temporal task [13, 14, 18] and the noisier representation of the perceived temporal interval in the memory [22, 25]. Thus, we considered it essential to isolate the brain’s passive perception of time from other active cognitive processes in the task by using rhythmic sequences.

Turgeon and colleagues [13] suggested that rhythmic sequences can remain intact even when single intervals may be impaired with age. This could be because rhythmic sequences automatically compel attention [26, 27], and thus make use of little controlled attentional resources. Nevertheless, these sequences are thought to evoke both sensory and motor representations [28], allowing distortions in the perceptual detection of temporal intervals to be seen if structural changes in the brain are the cause of impairments in temporal cognition in older adults.

### The present studies

The present studies tested whether the perception of time in older and younger adults differed when using rhythmic sequences to minimize the load of memory and attention involved in the task. In Study 1, we aimed to observe whether temporal perception differed in older adults compared to younger adults in both explicit and implicit tasks. Furthermore, Study 2 was added to explore whether the addition of a secondary task could impair the performance of older adults in the implicit task.

Out of the multitude of studies focusing on the dissociation between explicit and implicit timing and the evolution of temporal cognition in age, to our knowledge there is only one study that explored the combination of these two factors [11]. Our study is unique in the sense that we explore differences between the performances of older and younger adult groups in different rhythmic timing tasks, which are considered to recruit different structures from single intervals [5, 29]. We also deemed Bayesian modeling to be more appropriate for statistical analysis, for it can quantify evidence against the null hypothesis of no-difference [30].

Reported data and analysis scripts from all experiments in this study are publicly available on the Open Science Framework (https://osf.io/72a8b//).

## Study 1

Study 1 compared age-related differences in the perception of explicit and implicit timing. For the explicit task, we asked participants to perform a beat-discrimination task. This task was termed explicit for participants have to consciously process the temporal information presented in the sequence of beeps. Given the dissociated neural structures involved in processing of explicit and implicit time [1, 2, 31] and the correlation of higher cognitive functions with performance on explicit tasks [32], we predicted that even with the reduction of higher cognitive functions, older adults would have a higher threshold in the beat-discrimination task than younger adults.

In addition, we aimed to elucidate whether performance distinctions exist in the implicit oddball task in older and younger adults, since this may not require as much cognitive control. With a similar aim in mind, Droit-Volet, Lorandi, and Coull [11] assessed performance of older adults in an implicit task. However, in their attempt, they realized that older adults used a compensation mechanism that made up for possible age-related differences in the performance of implicit timing tasks, proving no differences in performance accuracy despite their possible difficulty. Therefore, the task used in this study was adapted from that of Marchant and Driver [33]. In this task, participants were asked to react to an oddball embedded in a regular or irregular sequence. Though they were not told to focus on the type of sequence (therefore considered implicit task in this study), reaction time, found in Marchant and Driver [33] was faster for oddballs hidden in a regular sequence of the presentation of visual disks than in an irregular sequence. Although the differences cannot be compared between the reaction time for older and younger adults since older adults tend to be slower, we were interested in seeing whether sensitivity to the oddballs decreases with increasing irregularity. Based on the two studies mentioned above, we hypothesized that there is no alteration in the processing of implicit timing in older adults and that they are able to take advantage of the hidden information embedded in the sequence of beeps to increase their sensitivity to the oddballs in regular sequences.

### Methods and procedures

#### Participants

To ascertain the number of subjects necessary to test the differences between older and younger adult groups in the five rhythmic sequences used in this study (see below for details), an a priori power analysis was conducted using G*Power3.1 [34] for a within-between interaction given repeated measures. We used a large effect size (*f* = .39), deemed appropriate based on the results of Droit-Volet and colleagues [11], an alpha of .05, and default values for correlation among repeated measures and non-sphericity correction. Results showed that a total sample of 14 was required to achieve a power of .95.

Given the uncertainty in the true effect size, we doubled the sample size. Fifteen young adults (Mean age = 21.9; SD = 2.67; female = 6, male = 9) and 15 older adults (Mean age = 71.1; SD = 4.02; female = 7, male = 8) participated in the study. Young adult participants were recruited from The University of Tokyo. The older adults were recruited from the Meguro Ward Silver Human Resource Center. Participants reported normal auditory sensitivity, and all older adults scored over 27 (Range = 27–30; Avg. 29.27) on the Mini-Mental State Examination (MMSE).

All subjects gave written informed consent in accordance with the Declaration of Helsinki. The protocol was approved by the institutional review boards of The University of Tokyo, and the subjects were given monetary awards for their participation.

#### Stimuli presentation

**Fig 1:**
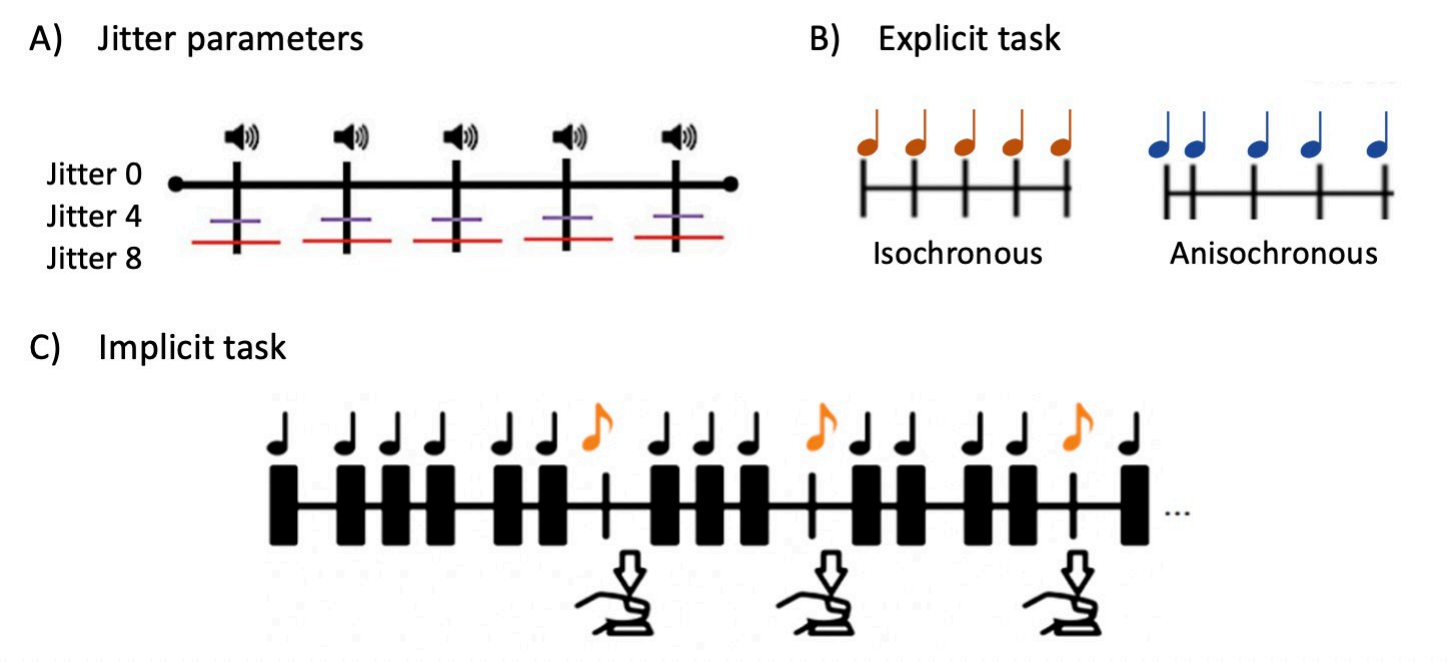
Methods and procedures for Study 1. A) Illustration of how the regularity of the sequence was jittered parametrically. Jitter zero, illustrated with the bold vertical line, refers to an isochronous sequence. The timing of these beeps was manipulated by moving the auditory beeps away from their center point by a certain percentage, and the greater the jitter, the more irregular the sequence. B) Procedure of the explicit task. Participants were required to judge whether the presented five beeps were isochronous (regular) or anisochronous (irregular). C) Procedure of the implicit task. Participants were required to press the button whenever they heard a shorter beep. The participants were instructed to ignore the regularity of the sequences in which the oddballs were embedded.

The stimuli used in the explicit and implicit timing tasks consisted of a presentation of auditory beep sequences. First, we prepared sequences of five beeps within two seconds or 50 beeps within 25 seconds for the explicit and implicit rhythmic tasks, respectively. For regular sequences, the interval between the beeps were of 400ms. The timing with which these beeps were played was jittered parametrically in order to create irregular sequences of beeps (Fig 1A). The onset of each beep in the sequence was jittered by 4.17%, 8.33%, 12.50%, 16.66%, 20.83%, 25%, 28.17%, and 33.33%, corresponding to one to eight-frame jitter (one frame = 16.7ms, corresponding to the 60Hz refresh rates of the monitor), respectively. Note that for each condition, for example, the eight-jitter condition, the perturbation of each beep was of negative eight, or eight frames or nothing. In the case of the explicit rhythmic task, we made sure that there was at least one of each negative and positive jitter, while the rest of the jitter were selected using the uniformly distributed pseudorandom integer function (randi) on MATLAB. On the implicit rhythmic task however, the jitter parameters used contained 16 non-jitters, 17 positive, and 17 negative jitters corresponding to each condition, and these were shuffled for each sequence.

These stimuli were presented at a listening level of 80dB using two speakers (Sanwa Supply: MM-SPL5BK) located on both sides of the CRT monitor (SONY-CPD E230) and a display resolution of 1024 by 768. Participants were asked to perform the task in a dark, soundproofed room (1.73m × 0.85m × 1.92m) facing the CRT monitor and to respond using a number keyboard. Stimuli presentation and data collection were done on a Mac Pro (mid-2010) with macOS Sierra using PsychportAudio and PsychToolbox extensions [35] on MATLAB 2017b.

#### Experiment 1A: Explicit task

The explicit timing task was a rhythm discrimination task. Five beeps (a stimulus of 3500Hz with a sampling rate of 6000Hz with a duration of seven milliseconds each) were presented in the span of approximately two seconds, and participants had to answer whether the auditory stimuli were presented in an isochronous or anisochronous manner. The anisochronous sequences were created by jittering the temporal intervals by one to eight frames of the otherwise constant inter-onset interval of 400ms.

Each trial started with a visual cue on the screen indicating that the trial would start, followed by an empty interval of 300 to 800ms preceding the auditory stimulus. We added the visual cue so that the participant could focus on the upcoming auditory sequence. After the presentation of the five beeps (Fig 1B), participants were instructed to press the “Tab” key of a number keyboard if the sequence was isochronous and the “Backspace” key if they perceived the sequence to be irregular.

In order to enhance the maximum efficiency of the task, we used the staircase method with a step-size of one jitter; participants spent most of their time in the jitter conditions close to their rhythmic threshold. Each block started with the most anisochronous sequences and worked its way down to isochrony over trials. Moreover, to avoid cognizance of the perceptually isochronous sequences appearing toward the end of the blocks, rather than in the beginning, we randomly added catch trials of regular sequences between the trials. The block terminated when there were eight reversions. The entire experiment lasted approximately 10 minutes, broken into six blocks so that the participants could take a break between the blocks if needed.

All participants performed a practice run with the experimenter outside the experiment room to make sure they had understood the task well, and they were asked to report whether they had any difficulty hearing the auditory stimulus. None of the participants reported hearing difficulties. Then, they moved to the soundproof room to proceed to the real experiment alone.

#### Experiment 1B: Implicit task

The implicit timing task was an oddball task, which was adapted from Marchant and Driver [33]. Instead of using visual stimuli, we kept the stimuli as identical as possible to the explicit task. As in the explicit experiment, the auditory beeps marked 400ms inter-onset intervals in the isochronous sequence, while the beeps in the irregular sequence were jittered by two, four, six, or eight frames. Unlike conventional practice, the pitch was not manipulated for the oddballs; instead, we altered the duration of the beeps. While the standard auditory stimuli were of 50ms in duration, the oddballs were presented for just 10ms. This form of oddball presentation was intended to avoid the automatic perceptual pop-up effect, such that participants were expected to focus on the sequence of the beeps implicitly; more importantly, however, this did not cause any perceptual difference in the rhythm of the sequence. Furthermore, oddballs were restricted from occurring within 1.5 sec of the initiation of the first beep and the last beep as well as from another oddball. In each trial (Fig 1C), participants were asked to listen to a sequence of 50 auditory beeps (80dB, 3500Hz tone with a sampling rate of 6000Hz), six of which were oddballs.

During the trial, participants were asked to press the “Enter” button of the number keyboard with their right index finger as quickly as they could when they heard the oddball. No specific instruction was given to attend to the type of sequence, unlike the explicit experiment.

All participants performed a practice run with the experimenter outside the experiment room to make sure they had understood the task well, and they were asked to report whether they had any difficulty hearing the auditory stimulus. None of the participants reported hearing difficulties. Then, they moved to the soundproof room to proceed to the real experiment alone. In the soundproof room, the participants performed a total of 30 trials, six for each sequence condition. The five sequence conditions were presented randomly within a block and there were 10 blocks total, between which participants could take a break if needed. The whole experiment lasted around 15 minutes.

The order of the two tasks were randomized such that eight of the old participants and seven of the young participants started with the explicit task first and then proceeded with the implicit task. The rest of the young and old participants started the experiment with the implicit task and then proceeded to the explicit task. The explanation of each task was given prior to the corresponding task so that the participants could take a break between each task as well.

### Results

#### Experiment 1A: Explicit task

To identify each participant’s auditory rhythmic threshold for each block, we calculated the averages of the last ten endpoints for each; this eliminated the early trials of the block where the participants were approaching their perceptual threshold. We considered the averages of the six blocks to be the average rhythmic threshold for each participant.

**Fig 2:**
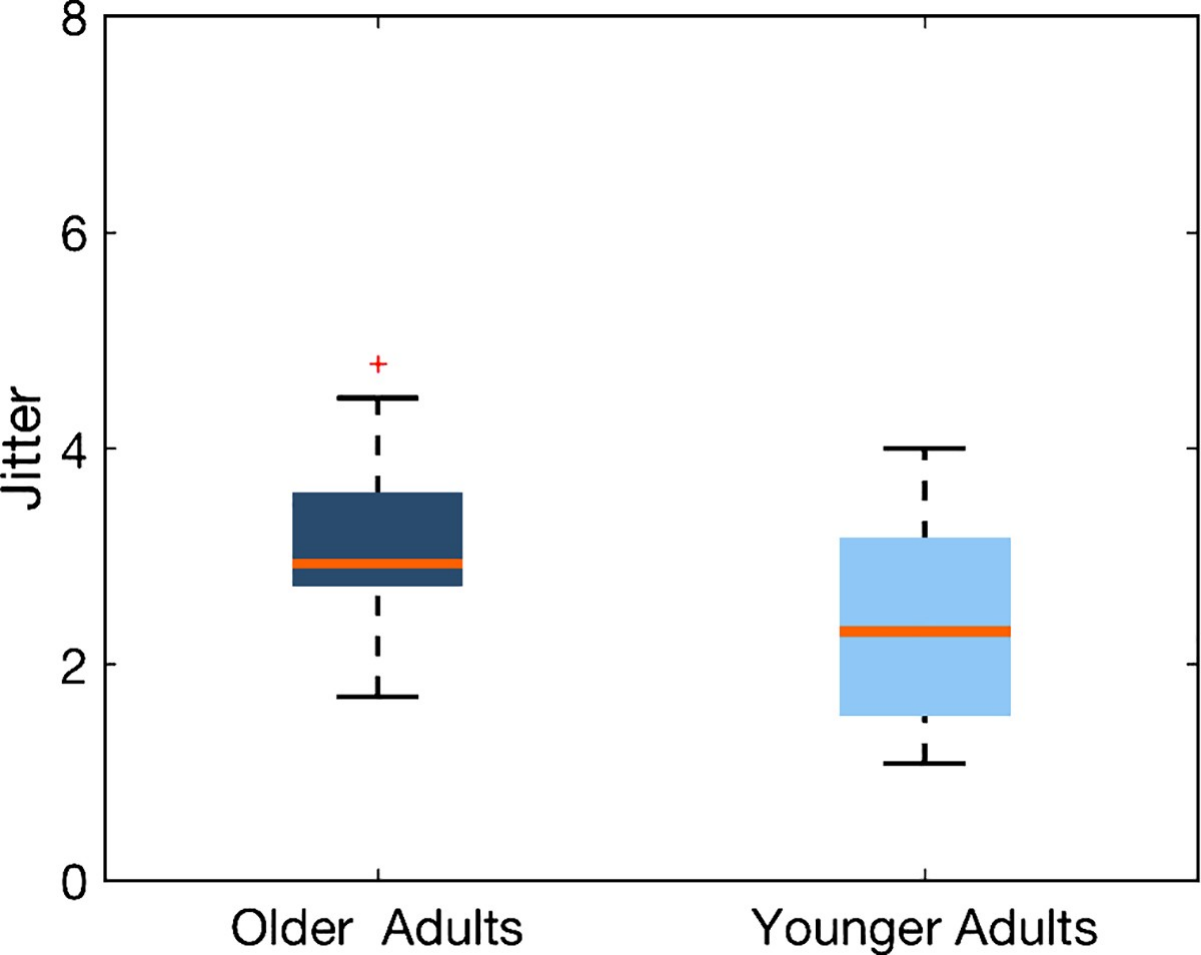
Group average of the threshold calculated from the explicit task. Data of older and younger adults are shown in dark shade and light shade, respectively. The central line indicates the median, and the edges of the box the inter-quartile range of 25^th^ and 75^th^ percentiles. The whiskers depicted by the dotted lines indicate the maximum and minimum values, excluding outliers (outliers are data points that are 1.5 times greater than the interquartile range). The further away the threshold is from zero, the more irregular sequences are perceived to be regular.

We hypothesized that older adults would have a higher threshold than younger adults based on the general notion that older adults perform worse than younger adults. Indeed, Fig 2 shows a boxplot of the average threshold of the younger and older participant groups, indicating that older adults had a higher threshold (*N* = 15; Mean threshold = 3.092; *SD* = 0.844) than younger adults (*N* = 15; Mean threshold = 2.381; *SD* = 0.864). The Bayes factor (*BF*_*10*_ = 4.381, error = 2.53e^−4^%; S1 Table) shows moderate evidence in favor of the alternative hypothesis, indicating that it is 4.381 times more likely for there to be a difference between the two age groups than no difference. These results suggest that older adults exhibited a higher threshold than did the younger adults: That is, the older adults perceived more anisochronous sequences to be “isochronous” than younger adults.

Also, to investigate whether older participants with a certain characteristic had a higher threshold, the Bayesian Pearson correlation was calculated between the threshold of each older individual, their age, and the raw MMSE scores. However, no significant correlations were found (Pearson’s *r* = −0.057; *BF*_*10*_ = 0.324), possibly due to an insufficient number of subjects.

Moreover, the sample size was determined based on the a priori analysis conducted using G*Power for repeated-measures ANOVA, which is more apt for the implicit task. Therefore, for the explicit task we also conducted a Bayesian robustness check on JASP [36] as well as a Bayesian predictive posterior check that generates 100 new simulations that test our null hypothesis. Results show that our moderate evidence was robust regardless of our prior distribution (S1 Fig) and that we had a power of 0.99.

#### Experiment 1B: Implicit task

*Reaction time*. The mean reaction time was calculated from the responses to the oddball. Initially, we calculated the average of the time between an oddball and the first button press. All the button presses that appeared two standard deviations (response time window) from the average of the mean reaction of each participant were removed and counted as “false alarm” responses. The average response time from the remaining button presses was recalculated to be the reaction time to be analyzed.

**Fig 3:**
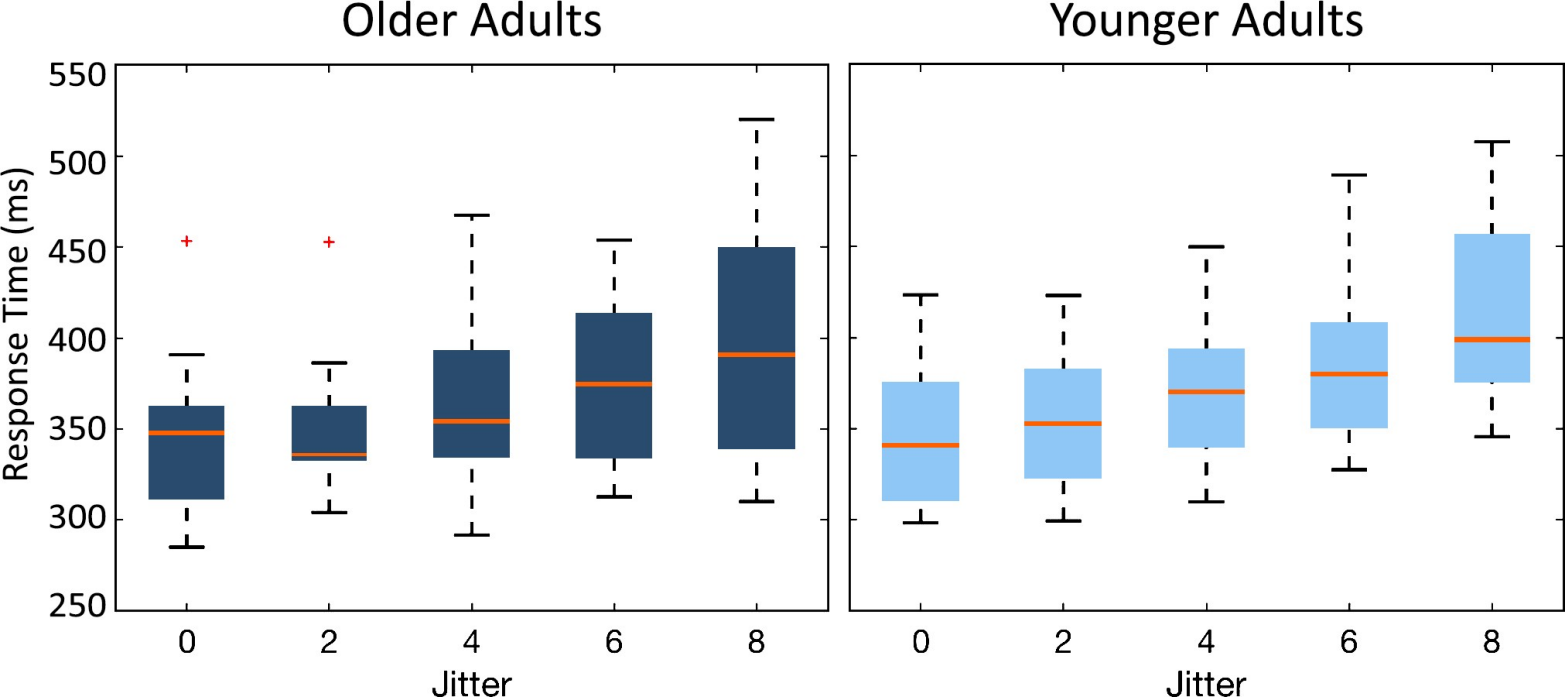
Reaction time of both older (dark shade) and younger (light shade) subjects to the oddballs located in a regular (0-jitter) and irregular (8-jitter) sequence. The central line indicates the median, and the edges of the box the interquartile range of 25^th^ and 75^th^ percentiles. The whiskers depicted by the dotted lines indicate the maximum and minimum values, excluding outliers (outliers are data points that are 1.5 times greater than the interquartile range).

Fig 3 illustrates the mean reaction time for the oddballs in both older and younger participant groups for each jitter sequence. A two-way Bayesian repeated measure ANOVA on mean response time revealed that the model including only the factor Jitter (*BF*_*10*_ = 7.170e^13^, error = 0.540%; S2 Table) received the most support from the data against the null model. This reveals moderate evidence for the main effect of Jitter (*BF*_*Inclusion*_ = 7.500 e^13^): That is, response time increased with increasing jitter. Results also revealed anecdotal support for the main effect of Age (*BF*_*Inclusion*_ = 0.492), which indicates that we cannot completely neglect the fact that older adults may have slower response times than younger adults. However, adding the factor of age decreases the model performance by 49%. Therefore, we cannot make a strong conclusion about the main effect of age; there does not seem to be enough evidence supporting differences between the performance of younger and older participant groups. In comparison to Age, the main effect of Jitter is much larger.

Our primary interest, however, was to see whether older adults benefitted from the implicit rhythmic information as younger adults did. More specifically, we predicted that older adults might not perform differently from younger adults when asked to respond to the oddballs in the different jittered sequences. There was substantial evidence supporting the null hypothesis for the interaction effect of “Jitter × Age” (*BF*_*Inclusion*_ = 0.070). These results indicate that older and younger adults benefited equally from the rhythmicity of the sequence in which the oddballs were embedded. In other words, they were able to benefit equally from the hidden temporal information in the rhythm of the sequences to find the oddball.

*Sensitivity*. Next, we calculated the sensitivity to the oddballs to determine whether there is evidence for sensitivity differences between older and younger adults. Sensitivity measures provide information about the signal and noise distribution in perception. “Hit” was defined to be the first response after the oddball stimulus occurring within the response time window. The hit rate, therefore, was calculated as the number of hits divided by the total number of oddballs presented across the five trials of each sequence condition. “False Alarm” was defined to be the sum of the number of responses prior to any presentation of an oddball, the second button press after the presentation of an oddball, and the button press occurring outside the response time window. We combined both hit-rates and false-alarm rates to obtain oddball detection sensitivity measures (d-prime).

**Fig 4:**
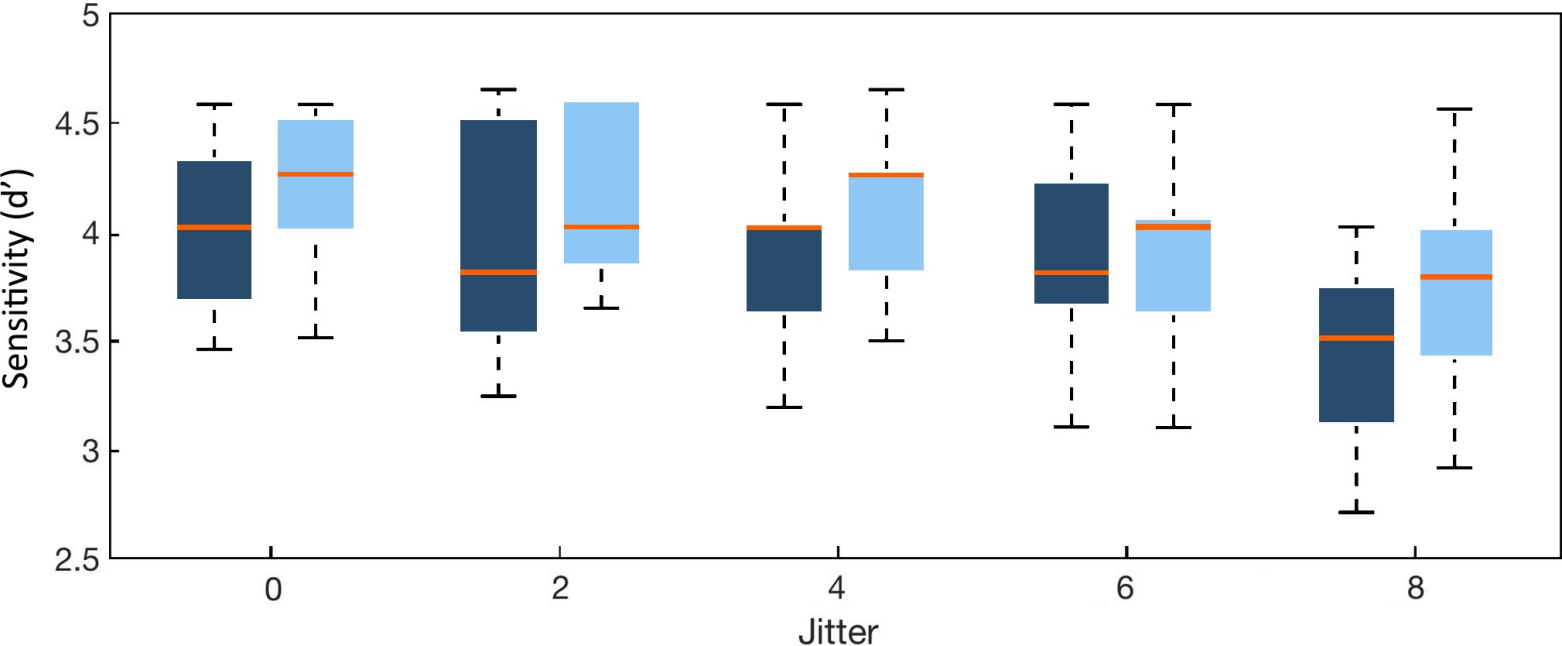
Measures of sensitivity to the oddballs in a regular (0-jitter) and irregular (8-jitter) sequence for older (dark shade) and younger (light shade) adult groups. The central line indicates the median, the edges of the box the inter-quartile range of 25^th^ and 75^th^ percentiles. The whiskers depicted by the dotted lines indicate the maximum and minimum values, excluding outliers (outliers are data points that are 1.5 times greater than the interquartile range).

Model comparisons using two-way Bayesian repeated-measure ANOVA on d-prime yield overwhelming evidence for the model containing the additive effect of Jitter and Age (*BF*_*10*_ = 39837.005, error = 1.362%; S3 Table) against the null hypothesis. Specifically, analysis of the effects shows strong evidence for the main effect of Jitter (*BF*_*Inclusion*_ = 35260.502) as well as anecdotal evidence for the main effect of Age (*BF*_*Inclusion*_ = 1.215). That is, in accordance with Marchant and Driver’s study [33], the sensitivity to oddballs increases as the sequence becomes increasingly regular (Fig 4). Moreover, the existence of anecdotal evidence of the main effect of Age reveals that older adults may be less sensitive to the oddballs overall than younger adults. Though the model containing only the effect of Jitter (*BF*_*10*_ = 32794.961, error = 0.542%; S2 Table) shows a close second in model performance, the model “Age + Jitter” outperforms the latter by approximately 21%. This number indicates that the factor of Age is likely to improve model performance, but again, we cannot make a strong conclusion about its contribution to the model.

Our main interest was the interaction effect between Age and Jitter; results showed moderate evidence supporting the null hypothesis (*BF*_*Inclusion*_ = 0.263). That is, though older adults may have less sensitivity overall to the oddballs, both age groups benefitted similarly from the implicit temporal information in the sequence of beeps.

### Discussion

The results of Study 1 show that while both explicit and implicit tasks utilized sequences of beeps that lower the attentional resources necessary to complete the task, age differences were observed in the explicit but not the implicit timing task. This statement holds even when cognitive factors, such as attention and working memory, were minimized in the tasks. Therefore, these results seem to confirm past studies stating differences in explicit and implicit mechanisms [1, 7–9].

## Study 2

Although the results of Study 1 confirm the dissociation of explicit and implicit timing, the extent to which higher cognitive factors such as attentional load were reduced it remains uncertain. As assumed by the attentional-gate model [37], all timing tasks recruit attentional resources, especially in the process of making a decision. To further explore the effects of attentional load and increased working memory demands, we included a task secondary to the implicit task, where age-related differences in the interaction between reaction time and jitter, and that of sensitivity and jitter were not observed.

Studies with younger adults have shown that rhythmic sequences are not vulnerable to a secondary task [26, 27]. However, under the Selective Attention Theory, which posits that humans only have a limited amount of attentional resources that must be distributed across tasks, it is possible that older adults may use more of these resources to compensate for the difficulty of the task demands. Hence, adding a secondary task may cause competition for insufficient attentional resources, having detrimental effects on the performance of older adults in the implicit timing task.

### Methods and procedure

#### Participants

Similar to Study 1, a priori power analysis was conducted using G*Power3.1 [34] to determine the necessary sample size for a within-between interaction given repeated measures, accounting for the two added memory load conditions for the five jitter conditions. We used a medium effect size (*f* = .25), an alpha of .05, and the default values for correlation among repeated measures and non-sphericity correction for this calculation. When the number of repeated measures was 10, results showed that a total sample of 20 was required to achieve a power of .95.

To have a comparable sample size to that of Study 1, participants were again composed of 15 young adults (Mean age = 22.2; *SD* = 2.14; female = 4, male = 11) and 15 older adults (Mean age = 73.6; *SD* = 3.33; female = 8, male = 7), eight of whom had participated in Study 1. Young adult participants were recruited from The University of Tokyo, and the older adults were recruited from the Third Generation Human Resource Center in Meguro-ward. Participants reported normal auditory sensitivity, and all older adults scored over 27 (Mean score = 29.3; *SD* = 0.96) on the Mini-Mental State Examination (MMSE).

All subjects gave written informed consent in accordance with the Declaration of Helsinki. The protocol was approved by the institutional review boards of the University of Tokyo, and the subjects were given monetary awards for their participation.

#### Stimuli presentation

The stimuli consisted of the presentation of a sequence of auditory beeps similar to that of the implicit task. We replicated the experimental set up as much as possible so as not to add any other variables that might interfere with the effects of memory load. However, due to the addition of two new conditions, the sequence of beeps utilized in the implicit task was reduced to 36 beeps containing three oddballs to avoid fatigue, especially in older participants.

#### Experiment 2: Implicit task with memory task

**Fig 5:**
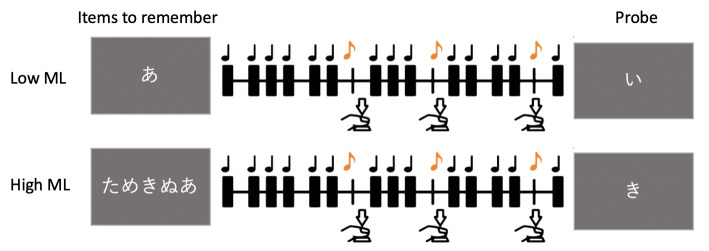
Procedure of the implicit task with a secondary memory task (an adaptation of the Sternberg’s paradigm). Participants were asked to remember a list of characters while reacting to the oddball. At the end of the trial, they answered whether the character on the screen was one that they had remembered. ML stands for memory load.

To manipulate the memory load (ML) of each task, we adopted the Sternberg item-recognition paradigm [38] as a secondary task to the implicit timing task (Fig 5). As in the previous implicit task, participants were instructed to respond as quickly as possible to the shorter beep (10ms) hidden in a regular or irregular sequence by pressing the “Enter” button on the number keyboard. Before the auditory sequence of beeps, the screen showed either one (lowML) or five (highML) Japanese characters (Hiragana) for two seconds. Participants were instructed to memorize the list of characters at the beginning of the trial. While the beeps were sounding and participants reacted to the oddball, they were expected to retain the memorized characters by repeating them in their heads. After the last beep of the sequence, a character appeared on the screen. Participants answered whether that item was in the list of characters presented at the beginning of the trial. The trial ended when the participant answered either “yes” by pressing the “Tab” button or “no” by pressing the “Delete” button.

A block consisted of six of these trials, with lowML and highML conditions mixed within the blocks. Participants were instructed to perform 10 of these blocks, completing 60 trials, six trials of each of the five jittered sequences times two, for each memory load. The entire experiment lasted around 30 minutes, and at the end of the task subjects rated the difficulty of the overall task for each of the ML conditions (low and highML).

### Results

#### Working memory task

The performance of the secondary working memory (WM) task was calculated for both low and high memory load conditions for both older and younger participant groups. Table 1 shows the descriptive results. We excluded two of the older participants from further analysis for they showed performance that was indicative of being caught off-guard for the lowML condition; One of the participants performed at a below-chance level (46.7%) in the lowML condition versus the 86.7% in the highML, and the other rated the lowML as more difficult than the highML (7:1, respectively) with a performance of 56.7% in the lowML and 93.3% in the highML. Three younger adults also rated the lowML as being harder than the highML condition: they explained that the surface simplicity of the task caused them to relax and underestimate its actual difficulty. However, as they had lower scores on the highML task than the lowML task, they were not excluded from the following analysis.

**Table 1:** Average difficulty rating (from one [easy] to seven [hard]) and accuracy score of the working memory task in Study 2 for older and younger adults.

		Low WM load		High WM load	
	n	Rating	Score	Rating	Score
**Older adults**	**13**	2.00	95.56%	3.62	95.18%
**Younger adults**	**15**	2.43	98.44%	3.27	95.5%

Note. Two of the older participants were excluded.

Bayesian repeated measures ANOVA revealed that though there was a main effect of Memory load (*BF*_*Inclusion*_ = 3.295) such that accuracy was lower when participants were asked to remember five characters, there was no evidence supporting main effect of Age (*BF*_*Inclusion*_ = 0.807) nor an interaction effect of “Memory Load × Age” (*BF*_*Inclusion*_ = 0.393).

#### Reaction time

With the addition of a dual-task in the implicit paradigm, we hypothesized that age-related differences would be more pronounced than in the results of the previous implicit task. After the exclusion of two older participants as detailed above, we again conducted Bayesian repeated-measures ANOVA and applied the same statistical analysis.

This time, the model including the additive factor of “Age + Jitter” outperformed the null model (*BF*_*10*_ = 6122.757, error = 1.473%; S4 Table). This model outperformed the only-Jitter model by a factor of 9.379.

**Fig 6:**
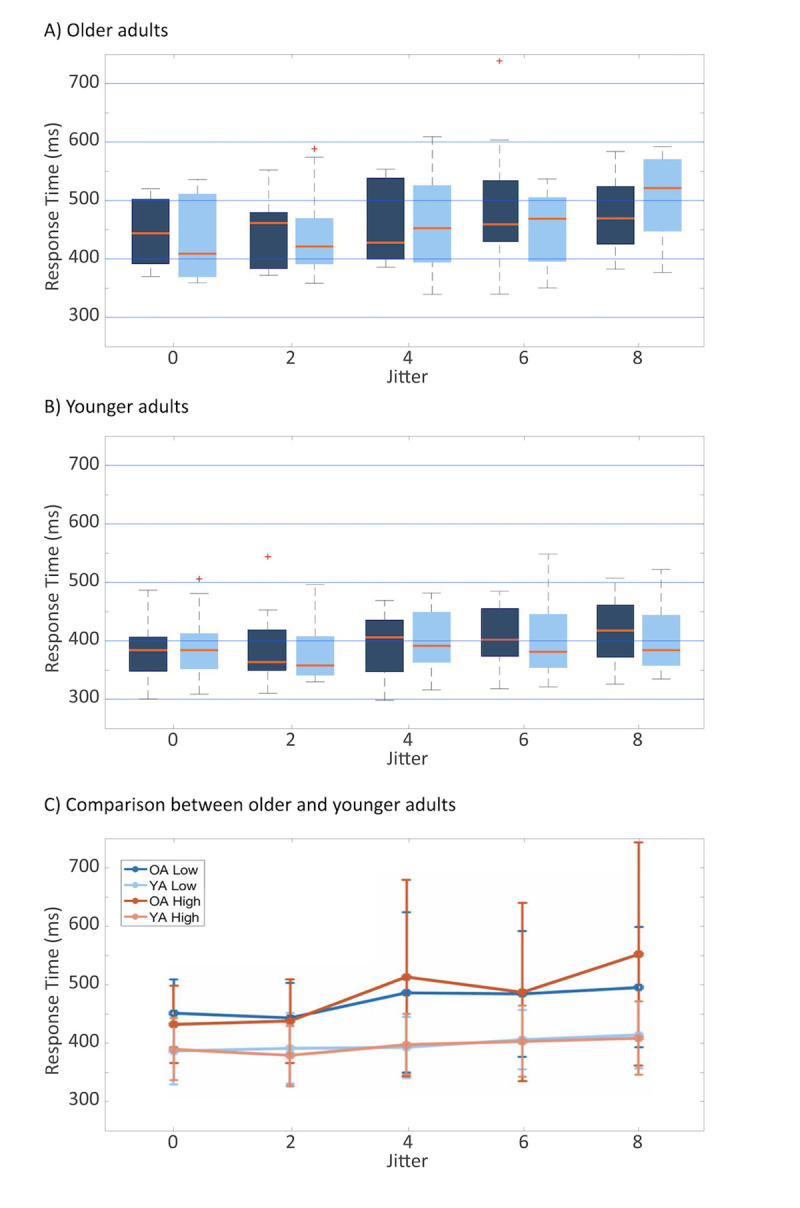
Reaction time for the implicit task in Study 2. The boxplots illustrate the reaction time of both older (A) and younger (B) subjects to the oddballs located in a regular (0-jitter) and irregular (8-jitter) sequence. The darker shades indicate the reaction time when the task involved lower (dark shade) and higher (light shade) memory loads. The central line indicates the median, the edges of the box the interquartile range of 25^th^ and 75^th^ percentiles. The whiskers depicted by the dotted lines indicate the maximum and minimum values, excluding outliers (outliers are data points that are 1.5 times greater than the interquartile range). The line plot (C) illustrates the trend of the reaction times of older (dark shade) and younger (light shade) participants. The solid and dotted lines indicate higher and lower memory loads, respectively. The error bars denote standard error.

The analysis of effect also supports this. While Jitter has the highest Bayes Factor (*BF*_*Inclusion*_ = 576.198), evidence supporting the main effect of Age (*BF*_*Inclusion*_ = 9.340) approaches the level of strong evidence. Therefore, we can conclude that while increasing jitter increased response times, as expected from Marchant and Driver [33], response times were also slower in general for older adults (Fig 6). Nevertheless, we did not find evidence supporting age-related differences in the use of rhythmic sequence information (*BF*_*Inclusion*_ = 0.938).

Contradictory to our hypothesis, we did not observe an effect of dual-task on the response time. That is, we thought that older adults’ response times would be greater under the highML condition than under lowML. Nonetheless, our results showed moderate evidence favoring the null hypothesis for the interaction effect of “Memory Load × Age” (BF_Inclusion_ = 0.283).

#### Sensitivity

**Fig 7:**
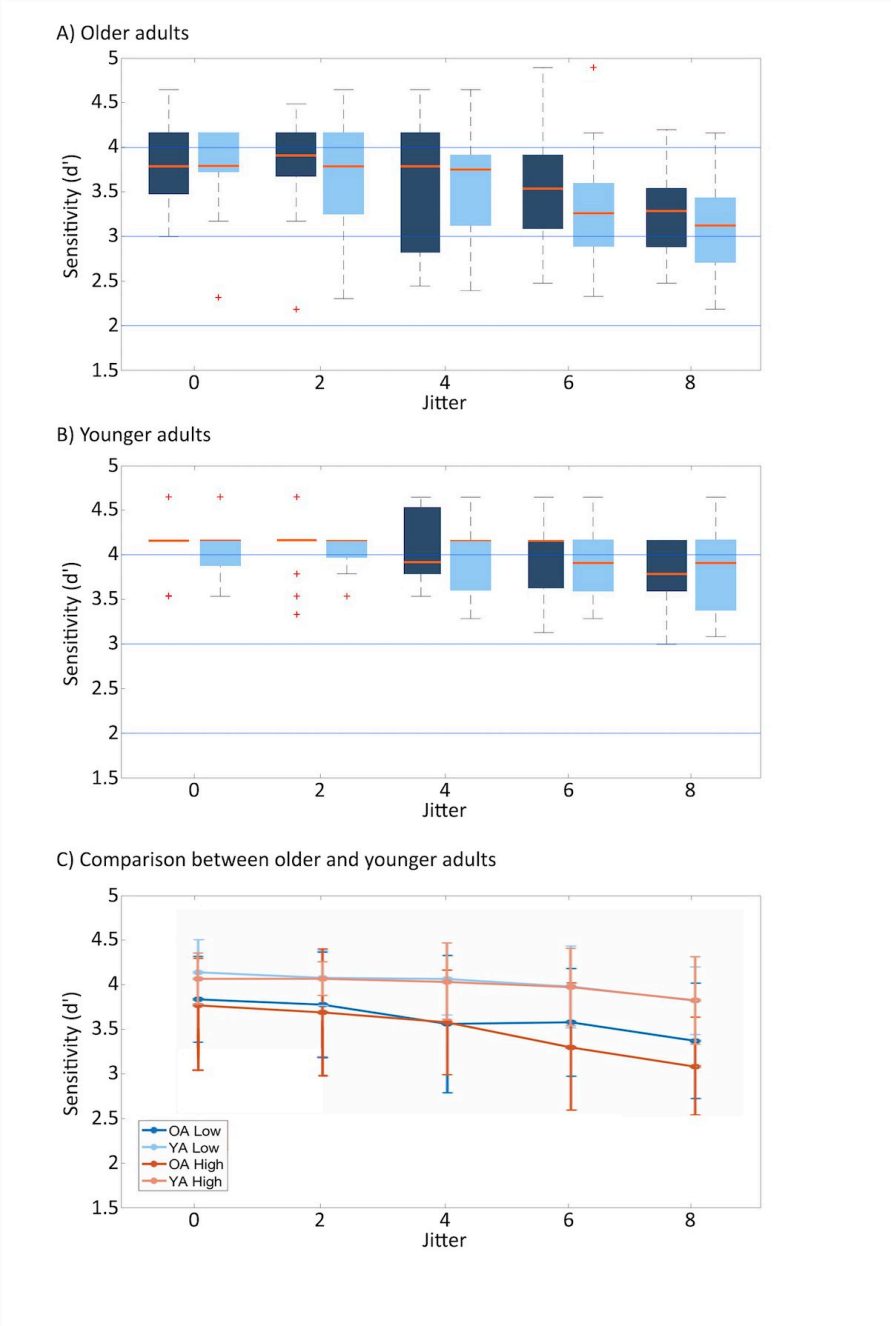
Sensitivity measures (d-prime) for the implicit task in Study 2. The boxplots illustrate sensitivity (Study 2) to the stimulus of both older (A) and younger (B) subjects to the oddballs located in a regular (0-jitter) and irregular (8-jitter) sequence. The darker shades indicate the sensitivity when the task involved lower (dark shade) and higher (light shade) memory loads. The central line indicates the median, the edges of the box the interquartile range of 25^th^ and 75^th^ percentiles. The whiskers depicted by the dotted lines indicate the maximum and minimum values, excluding outliers (outliers are data points that are 1.5 times greater than the interquartile range). The line plot (C) illustrates the trend of the sensitivity measures of older (dark shade) and younger (light shade) participants. Error bars denote standard error. The solid and dotted lines indicate high and low memory loads, respectively.

Similar to the previous implicit task, we hypothesized that older adults might have a higher level of noise than younger adults, causing decreased sensitivity to the oddballs. The results of Bayesian repeated-measures ANOVA showed that this was also the case: The best performing model against the null model was that of “Jitter + Age” (*BF*_*10*_ = 300415.904, error = 2.423%; S5 Table), which constitutes strong evidence against the null model. Indeed, the analysis of effects showed a robust main effect for Jitter (*BF*_*Inclusion*_ = 5530.860) and Age (*BF*_*Inclusion*_ = 58.296). However, we found moderate evidence suggesting no difference in the way the two age groups benefitted from the regularity of the sequence (*BF*_*Inclusion*_ = 0.130). In other words, we replicated the study of the previous implicit task, such that both subject groups’ sensitivity decreased with increasing jitter and internal noise increased with age (Fig 7).

We were particularly interested to see whether older adults had more difficulty in the timing task when there was increased demand in attentional resources. Results suggest, however, that there is moderate evidence supporting the null hypothesis for the interaction effect of “Memory Load × Age” (*BF*_*Inclusion*_ = 0.317). That is, memory load did not affect the two age groups differently.

### Discussion

Study 2 tested the effect of a dual-task on the implicit task used in Study 1 to see whether it is possible to magnify age-related differences with increasing attentional load. Previous studies have shown that the rhythmic sequences are resistant to increased attentional demands in younger adults [26] but not in older adults. Given that older adults have compromised cognitive capacity for demanding tasks, we hypothesized that more intricate tasks could imperil the perception of rhythmic sequences. Indeed, unlike in Study 1, older adults had slower response times than younger adults. Hence, although our results were insufficient to argue that memory load has an effect on the performance of older adults, there was a trend in the data suggesting a deterioration of general performance of older adults in the implicit timing task when the task involved a higher memory load.

## General discussion

In the present studies, we investigated the age-related differences in explicit and implicit tasks regarding the perceptual processing of time. Study 1 was conducted to investigate whether there are age-related differences in temporal processing of explicit timing and implicit timing when the effects of other cognitive factors like memory and attention, as well as motoric components, are suppressed. In Study 2, we explored whether increased cognitive load in the task jeopardizes the performance of older adults in the implicit task.

### Aging and explicit timing

Our study showed that older adults had higher beat-discrimination thresholds in the explicit task. We speculate that, like in people with PD [7], higher thresholds in older adults may be due to the decline of dedicated structures of temporal processing of explicit but not implicit time. As mentioned earlier, the basal ganglia are known to be involved in the processing of explicit time [1]. It is also said that activity in the basal ganglia is crucial in the encoding of temporal intervals [39] and responds more strongly when presented with a regular than an irregular sequence [40–42]. Therefore, it makes sense that older adults may have a harder time differentiating regular beats from irregular ones if they do have deficits in the dopaminergic circuit [43] and hence a slower internal clock.

Moreover, the higher threshold in older adults persisted even when attentional and memory components were minimized in the tasks. This result seems consistent with the fact that they may have a slower internal clock in the network involved in the processing of explicit temporal information, irrespective of attentional and memory components. The reduction of memory and attentional load, therefore, seems not to have been enough to suppress the age-related differences in the performance of the explicit task in older adults, as they performed worse than the younger group.

Nonetheless, we cannot conclude whether the impoverished performance was due to less accuracy in time perception of older adults, for it might have been due to the distorted representations of these rhythmic sequences in memory [39] or the increased level of perceptual noise [11]. More specifically, the explicit task may have required retention of the five beeps to judge whether the beat was isochronous. Along these lines, it is known that memory of higher jitter conditions declines more for more regular sequences [44]. This suggests that the task of maintaining the sequence in working memory might have caused a tendency toward regularity, thus leading to higher thresholds in older adults.

Furthermore, it is possible that rather than impoverished accuracy, precision is reduced with the noisier temporal perception of older adults. In this explicit study, our task didn’t allow us to dissociate accuracy from precision. However, higher threshold in this task may be a result of decreased precision. In fact, Droit-Volet and colleagues [11] have recently shown that older adults have more noise in explicit timing despite preserved accuracy. Thus, the higher threshold observed in older adults in the explicit task may be indicative of a noisier clock rather than a slower pacing of the internal clock.

In relation to this, the beat-discrimination threshold in the explicit task were not correlated to the MMSE scores. The MMSE, however, is not a precise measure of the actual memory and attention ability; instead, it is a general cognitive test. Thus, we only used it as a criterion for participation in this study. Previous studies have shown that performance on timing tasks is correlated with standardized cognitive test scores [11, 45], and in specific, Droit-Volet and colleagues [11] found that the noise they found in the perception of explicit timing of single intervals was correlated with the ability of attentional control. Therefore, even though we did not find a direct correlation between MMSE and the threshold of older adults, a reduction in cognitive ability might somehow be related to the higher thresholds of the explicit task.

### Aging and reaction time for implicit timing

Our study showed that implicit processing of time remains intact with age when utilizing rhythmic sequences. As in previous studies with younger adults [9, 33], older participants benefitted equally from the temporal information embedded in the sequences used for the implicit task such that response times were faster for a regular sequence than an anisochronous sequence. This result is also along the lines of Droit-Volet, Lorandi and Coull [11] for they showed that older adults, though employing different compensation mechanisms in the implicit task, performed well with the implicit timing task compared to younger adults. Similar performance between age groups is an indication that older adults can accurately perceive and employ implicit rhythmic information.

Neural mechanisms involved in temporal processing of explicit and implicit tasks can explain why the performance of older adults in the implicit task is intact when that of the explicit task is not. The effects aging on the basal ganglia [43] has constantly been a focus in the rhythmic perception [7, 13, 41]. Unlike in the explicit processing of time, implicit processing of temporal structures employs different neural structures that are unrelated to processing in explicit timing tasks [1, 2, 7, 43, 46]. In specific to this task, sensory cortices seem to be important in processing oddball information embedded in regular sequences [33, 40]. Therefore, older adult’s perception of rhythmic timing may not be completely compromised, rather, implicit processing of temporal information is intact despite reduced performance in explicit timing.

However, as mentioned earlier, worse performance on the explicit task may not necessarily correspond to a decreased speed of the clock. It is possible that explicit tasks recruit more attentional control and thus have noisier perception [11], while performance in the implicit tasks did not show much age related differences in terms of reaction times to the oddball. One thing for sure is that the conception of slower pacing clock speed of older adults is not applicable in the implicit temporal processing for accuracy is preserved with age.

### Memory load and implicit timing

To test whether the lack of age-related differences in the implicit task could be maintained with increasing memory load, we added a secondary task to the implicit task. We had hypothesized that older adults may be compensating for the task difficulty, especially in the decision-making process, in ways that younger adults may not need to, and the additional secondary task in a non-temporal domain might compete with the primary timing task. Our results were indicative of no dual-task effect on the performance of both older and younger adults, replicating the study of De la Rosa and colleagues [26] using younger adults, in both reaction time and sensitivity measures. However, this seems unlikely, especially for older adults, for the attentional resources required for the memory task and cognitive demands alter their performance on other tasks [15].

Similarly, Henry and colleagues [14] found age-related differences in neural entrainment by rhythmic sequences for a task that required higher attentional resources than one that did not. Although there was insufficient evidence to support age-related differences in the use of information embedded in the rhythmic sequences, the trend in our results suggests that there was an average of slower response times, especially in the more anisochronous sequences of older adults under conditions of higher working memory load. Thus, we still question the likelihood that memory does not have an impact on the temporal processing of implicit time.

It may in fact be possible that memory task used here was not a good dual task paradigm. Sternberg paradigm [38] was used here to disturb the processing of the rhythmic auditory stimulus because phonological working memory recruits brain regions in the auditory cortex [47]. Yet, the Sternberg paradigm [38] used here might have been insufficient to test the contribution of memory load on the timing tasks even though it has been tested to show efficacy in several studies. We note that as we tested Japanese participants, we changed the items to Japanese characters; in doing so, we might have made the task easier. Unlike lists of letters, random Japanese characters form pseudo-words that may in fact only count as one item. Thus, differences in the load of lowML containing one character and highML containing five characters might have been small. Sternberg’s paradigm in Japanese, therefore, may have recruited attentional loads that were not too obviously different.

Nonetheless, in the task involving higher cognitive demands (Study 2), we found that reaction times were different across age groups, unlike in the single implicit task (Study 1). Our moderate evidence suggests that older adults’ response times were slower than those of younger adults, a result we had not observed in the previous implicit task, which did not involve a secondary task. It is possible that although there were no differences between high and low memory load conditions, the simple addition of a memory task enlarges age-related differences in reaction time.

Greater reaction times might not only be due to motor delay but might also be indicative of the decision-making process: A slow reaction time may indicate uncertainty as to whether an oddball was heard. Zanto and colleagues [48] found neuropsychological evidence stating that when task demands were high, older adults displayed delays in response times and P300, a neural signal that is known to be involved in decision-making. On the other hand, they did not observe age-related behavioral and neural differences when task demands were low. Hence, although we did not find differences within this task due to minimal differences under the conditions of Sternberg’s paradigm, asking the participants to remember lists of characters led to age-related differences in overall response times that were not observed in the implicit task without a secondary task.

It is possible that the slowing of reaction time in older adults is also due to limited attentional resources dedicated to processing time. Under the Selective Attention Theory, one only has a limited amount of attentional resources, which are known to be much more limited in older adults [49]. Perhaps the dual-task requires attentional resources of which older adults have relatively less. The automaticity of the allocation of attention in rhythmic tasks may require sufficiently small attentional resources of younger adults for their performance not to be altered by the dual-task, but not for the older cohort. Thus, we suggest that older adults may be recruiting compensation mechanisms and consuming more of their attentional resources to try to perform as well as their younger cohorts.

### Aging and noise in implicit tasks

In our study, the decreased sensitivity measures illustrated the involvement of noise in the perception of implicit rhythmic timing in older adults. The involvement of noise in the perception of implicit rhythmic timing in older adults was manifested in decreased sensitivity measures than in the younger age group in both the single and dual implicit tasks. Lower sensitivity is indicative of higher noise, and the fact that older adults have noisier perception has been previously demonstrated [11, 13, 22, 25, 50–52]. Our study further adds that the noisiness of temporal perception in older adults persists even when using implicit processing of rhythmic sequences that require less attentional control [26, 27]. However, this does not contribute to lowering the accuracy in the performance in the implicit task.

In both these studies, we also saw that sensitivity measures increased with increasing regularity, irrespective of age. That is, regardless of the increased level of noise, older adults seem to have benefitted from the regularity of the sequence in such a way that there was not enough evidence to conclude that they performed differently from younger adults.

Despite older adults benefitting from the regularity of the sequence equally as their younger counterparts, evidence supports that older adults had increased levels of noise compared with younger adults. Even with the lack of a dual-task effect in rhythms in studies using university students [26, 27], our study shows that older cohorts have increased level of noise when implicit task is conducted in a parallel manner with a memory task. This additional memory load may have recruited more controlled attention of older adults in order to perform as well as the younger adults on the reaction time task. Whereas, it would not be statistically correct to conclude that age differences were more prominent in the implicit task with a secondary task than without it, we can conclude in general that there is an increased level of noise in older age groups despite the use of rhythmic sequences.

## Conclusions

In this study, we used Bayesian statistical analysis to explore whether any age-related differences exist in the perception of explicit and implicit rhythmic timing sequences when we reduced higher cognitive demands of the task. We expected to see differences in explicit and implicit timing such that older adults’ performance on an implicit timing task remains intact while explicit timing performance differed with age, despite the reduced level of cognitive demands. Given our data, we argued that when task difficulty is adequately reduced, both timing mechanisms are unimpaired in older adults. However, the performance of older adults on the explicit task seemed to show substantially more noise than occurred in the implicit tasks. Furthermore, we found that increasing cognitive demands in the implicit timing task magnified evidence for age-related differences in performance. Thus, though rhythmic sequences may reduce age-related differences in the perception and processing of time, the attentional control required by explicit and dual tasks may result in expanded differences with age.

### Limitations and further research

This study was purely behavioral, exploring age-related differences in timing performance; hence, we cannot make strong arguments about the basal ganglia or other brain structures whose performance may decline with age. Nonetheless, the results shown here can provide a starting point for future exploration of rhythmic timing mechanisms and the brain.

## Supporting information

S1 Fig Robustness check for explicit task (Study 1). The Bayesian Independent Samples T-test analysis was performed using JASP with the threshold (V2) and age as the dependent and grouping variables.
(PDF)

S1 File (DOCX)

S1 Table Results for the explicit task (Study 1). The Bayesian Independent Samples T-test analysis was performed using JASP with the threshold (V2) and age as the dependent and grouping variables.
(PDF)

S2 Table Model comparison results for each participant’s response time for the implicit task (Study 1). Bayesian repeated-measures ANOVA was performed using JASP. The variables age group and jitter were considered to be a between subject factor and repeated measures factor, respectively.
(PDF)

S3 Table Model comparison results for each participant’s sensitivity measures (d-prime) for the implicit task (Study 1). Bayesian repeated-measures ANOVA was performed using JASP. The variables age group and jitter were considered to be a between subject factor and repeated measures factor, respectively.
(PDF)

S4 Table Model comparison results for each participant’s response times in the implicit task with working memory conditions (Study 2). Bayesian repeated-measures ANOVA was performed using JASP. While the age group was considered to be a between subject factor, memory load and jitter conditions were specified as factors of repeated measures.
(PDF)

S5 Table Model comparison results for each participant’s sensitivity measures (d-prime) in the implicit task with working memory conditions (Study 2). Bayesian repeated-measures ANOVA was performed using JASP. While the age group was considered to be a between subject factor, memory load and jitter conditions were specified as factors of repeated measures.
(PDF)
