# Supplementary material for: Older adults preserve accuracy but not precision in explicit and implicit rhythmic timing
Source: PLoS One. 2020 Oct 19;15(10):e0240863. doi: 10.1371/journal.pone.0240863 (PMC7571673; doi:10.1371/journal.pone.0240863)

S1 Figure. Robustness check for Explicit task (Study 1). The Bayesian Independent Samples T-test analysis was performed using JASP with the threshold (V2) and age as the dependent and grouping variables.

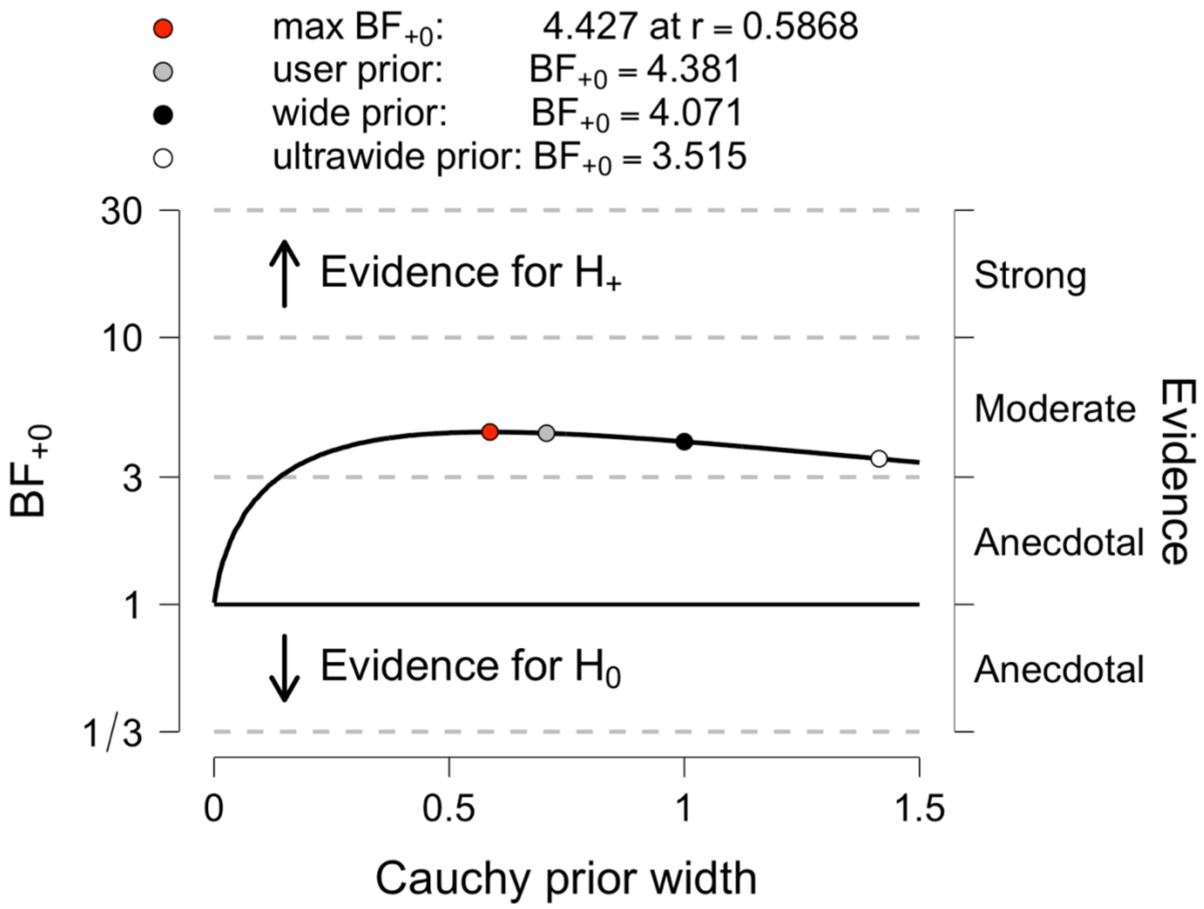

Supplement: S1 Fig — The Bayesian Independent Samples T-test analysis was performed using JASP with the threshold (V2) and age as the dependent and grouping variables. (PDF) [file pone.0240863.s001.pdf]
