# Supplementary material for: Older adults preserve accuracy but not precision in explicit and implicit rhythmic timing
Source: PLoS One. 2020 Oct 19;15(10):e0240863. doi: 10.1371/journal.pone.0240863 (PMC7571673; doi:10.1371/journal.pone.0240863)
Supplement: S1 File — (DOCX) [file pone.0240863.s002.docx]

Supplementary Material 1: Analysis for the explicit task (Study 1) done using JASP with the threshold and age as the dependent and grouping variables, respectively. A) Bayesian Independent Samples T-test with V2 as the threshold. B) Robustness check.

A)

| **Bayesian Independent Samples T-Test** | | | | | |
| --- | --- | --- | --- | --- | --- |
|  | | **BF₊₀** | | **error %** | |
| Threshold |  | 4.381 |  | ~ 2.531e -4 |  |
|  | | | | | |
| *Note.*  For all tests, the alternative hypothesis specifies that group *Old* is greater than group *Young* . | | | | | |

B)

**Bayes Factor Robustness Check**


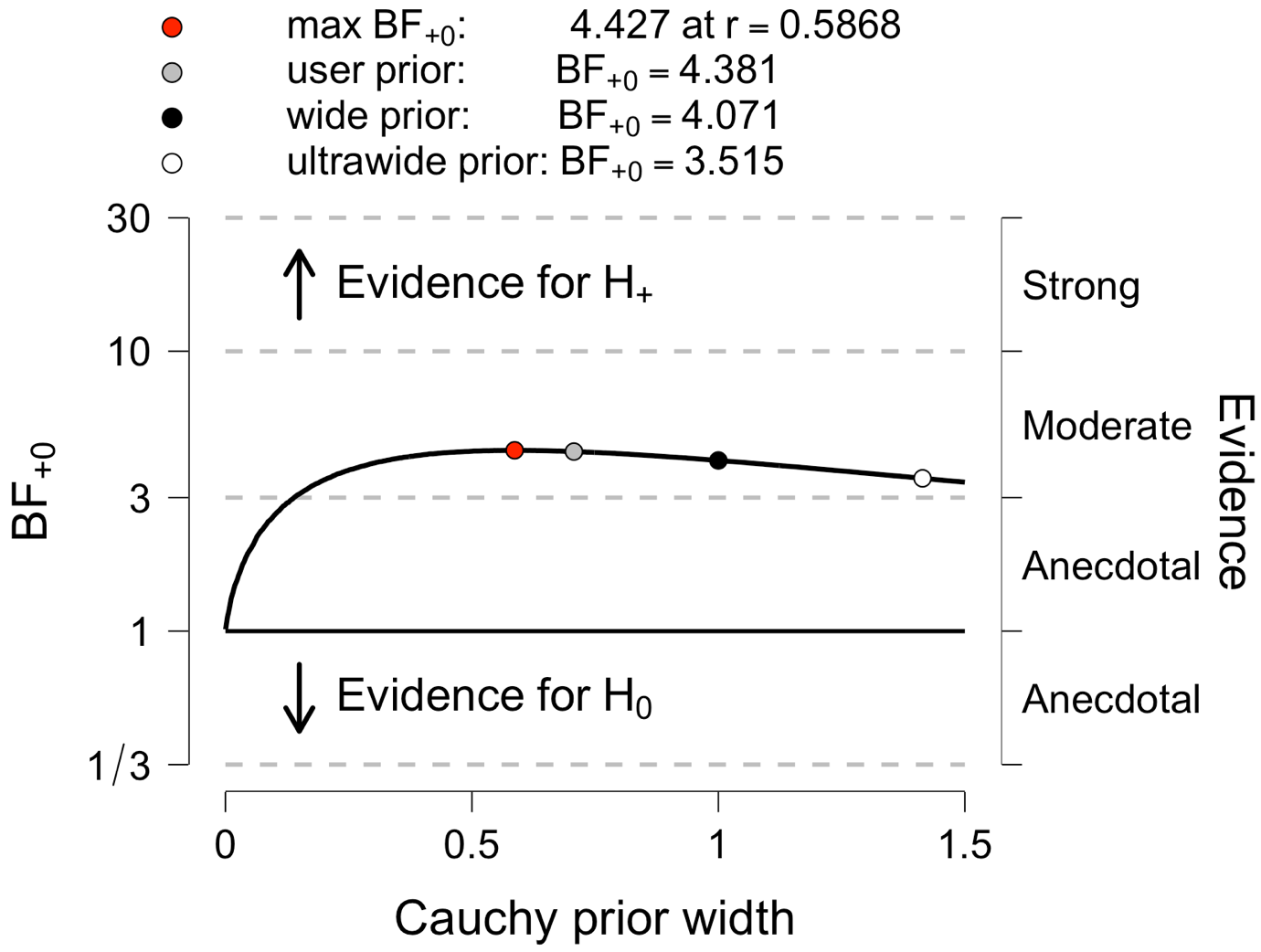


Supplementary Material 2: Bayesian repeated-measures ANOVA using JASP for the model comparison for each participant’s response time in the implicit task (Study 1). The variables age group and jitter were considered to be a between subject factor and repeated measures factor, respectively.

| **Model Comparison** | | | | | | | | | | | | | | | |  |
| --- | --- | --- | --- | --- | --- | --- | --- | --- | --- | --- | --- | --- | --- | --- | --- | --- |
| **Models** | **P(M)** | | | **P(M\|data)** | | | **BF _M_** | | | **BF _10_** | | | **error %** | | |  |
| Null model (incl. subject) |  | 0.200 |  | | 9.137$e^{-15}$ |  | | 3.655$e^{-14}$ |  | | 1.000 |  | |  |  | |
| Jitter |  | 0.200 |  | | 0.655 |  | | 7.597 |  | | 7.170$e^{13}$ |  | | 0.540 |  | |
| Age group |  | 0.200 |  | | 3.896$e^{-14}$ |  | | 1.558$e^{-14}$ |  | | 0.426 |  | | 0.659 |  | |
| Jitter + Age group |  | 0.200 |  | | 0.322 |  | | 1.903 |  | | 3.528$e^{13}$ |  | | 3.426 |  | |
| Jitter + Age group + Jitter ✻ Age group |  | 0.200 |  | | 0.023 |  | | 0.092 |  | | 2.469$e^{12}$ |  | | 6.115 |  | |
|  | | | | | | | | | | | | | | | |  |
| *Note.*  All models include subject. | | | | | | | | | | | | | | | |  |

Supplementary Material 3: Bayesian repeated-measures ANOVA using JASP for the model comparison for each participant’s sensitivity measures (d-prime) in the implicit task (Study 1). The variables age group and jitter were considered to be a between subject factor and repeated measures factor, respectively.

| **Model Comparison** | | | | | | | | | | | |
| --- | --- | --- | --- | --- | --- | --- | --- | --- | --- | --- | --- |
| **Models** | | **P(M)** | | **P(M\|data)** | | **BF _M_** | | **BF _10_** | | **error %** | |
| Null model (incl. subject) |  | 0.200 |  | 1.203$e^{-5}$ |  | 4.813$e^{-5}$ |  | 1.000 |  |  |  |
| Age group |  | 0.200 |  | 1.275$e^{-5}$ |  | 5.101$e^{-5}$ |  | 1.060 |  | 1.607 |  |
| Jitter |  | 0.200 |  | 0.395 |  | 2.607 |  | 32794.961 |  | 0.542 |  |
| Age group + Jitter |  | 0.200 |  | 0.479 |  | 3.682 |  | 39837.005 |  | 1.362 |  |
| Age group + Jitter + Age group  ✻  Jitter |  | 0.200 |  | 0.126 |  | 0.577 |  | 10475.853 |  | 3.070 |  |
|  | | | | | | | | | | | |
| *Note.*  All models include subject. | | | | | | | | | | | |

Supplementary Material 4: Bayesian repeated-measures ANOVA using JASP for the model comparison for each participant’s response times in the implicit task with working memory conditions (Study 2). While the age group was considered to be a between subject factor, memory load and jitter conditions were specified as factors of repeated measures.

| **Model Comparison** | | | | | | | | | | | |
| --- | --- | --- | --- | --- | --- | --- | --- | --- | --- | --- | --- |
| **Models** | | **P(M)** | | **P(M\|data)** | | **BF _M_** | | **BF _10_** | | **error %** | |
| Null model (incl. subject) |  | 0.053 |  | 6.569$e^{-5}$ |  | 0.001 |  | 1.000 |  |  |  |
| Memory Load |  | 0.053 |  | 1.033$e^{-5}$ |  | 1.859$e^{-4}$ |  | 0.157 |  | 3.678 |  |
| Jitter |  | 0.053 |  | 0.043 |  | 0.807 |  | 652.826 |  | 0.804 |  |
| Memory Load + Jitter |  | 0.053 |  | 0.007 |  | 0.128 |  | 107.344 |  | 5.892 |  |
| Memory Load + Jitter + Memory Load  ✻  Jitter |  | 0.053 |  | 4.245$e^{-4}$ |  | 0.008 |  | 6.462 |  | 2.026 |  |
| Age group |  | 0.053 |  | 7.291$e^{-4}$ |  | 0.013 |  | 11.100 |  | 15.798 |  |
| Memory Load + Age group |  | 0.053 |  | 9.719$e^{-5}$ |  | 0.002 |  | 1.479 |  | 2.338 |  |
| Jitter + Age group |  | 0.053 |  | 0.402 |  | 12.111 |  | 6122.757 |  | 1.473 |  |
| Memory Load + Jitter + Age group |  | 0.053 |  | 0.064 |  | 1.223 |  | 968.145 |  | 5.967 |  |
| Memory Load + Jitter + Memory Load  ✻  Jitter + Age group |  | 0.053 |  | 0.004 |  | 0.080 |  | 67.426 |  | 6.706 |  |
| Memory Load + Age group + Memory Load  ✻  Age group |  | 0.053 |  | 2.388$e^{-5}$ |  | 4.298$e^{-4}$ |  | 0.363 |  | 1.716 |  |
| Memory Load + Jitter + Age group + Memory Load  ✻  Age group |  | 0.053 |  | 0.018 |  | 0.329 |  | 273.222 |  | 4.746 |  |
| Memory Load + Jitter + Memory Load  ✻  Jitter + Age group + Memory Load  ✻  Age group |  | 0.053 |  | 0.001 |  | 0.021 |  | 17.400 |  | 2.869 |  |
| Jitter + Age group + Jitter  ✻  Age group |  | 0.053 |  | 0.381 |  | 11.095 |  | 5804.935 |  | 1.177 |  |
| Memory Load + Jitter + Age group + Jitter  ✻  Age group |  | 0.053 |  | 0.057 |  | 1.083 |  | 863.955 |  | 1.895 |  |
| Memory Load + Jitter + Memory Load  ✻  Jitter + Age group + Jitter  ✻  Age group |  | 0.053 |  | 0.004 |  | 0.070 |  | 58.936 |  | 1.829 |  |
| Memory Load + Jitter + Age group + Memory Load  ✻  Age group + Jitter  ✻  Age group |  | 0.053 |  | 0.016 |  | 0.296 |  | 246.009 |  | 3.180 |  |
| Memory Load + Jitter + Memory Load  ✻  Jitter + Age group + Memory Load  ✻  Age group + Jitter  ✻  Age group |  | 0.053 |  | 0.001 |  | 0.020 |  | 16.778 |  | 2.294 |  |
| Memory Load + Jitter + Memory Load  ✻  Jitter + Age group + Memory Load  ✻  Age group + Jitter  ✻  Age group + Memory Load  ✻  Jitter  ✻  Age group |  | 0.053 |  | 1.710$e^{-4}$ |  | 0.003 |  | 2.603 |  | 2.101 |  |
|  | | | | | | | | | | | |
| *Note.*  All models include subject. | | | | | | | | | | | |

Supplementary Material 5: Bayesian repeated-measures ANOVA using JASP for the model comparison for each participant’s sensitivity measures (d-prime) in the implicit task with working memory conditions (Study 2). While the age group was considered to be a between subject factor, memory load and jitter conditions were specified as factors of repeated measures.

| **Model Comparison** | | | | | | | | | | | |
| --- | --- | --- | --- | --- | --- | --- | --- | --- | --- | --- | --- |
| **Models** | | **P(M)** | | **P(M\|data)** | | **BF _M_** | | **BF _10_** | | **error %** | |
| Null model (incl. subject) |  | 0.053 |  | 1.891$e^{-6}$ |  | 3.403$2e^{-5}$ |  | 1.000 |  |  |  |
| Memory Load |  | 0.053 |  | 6.528$e^{-7}$ |  | 1.175$e^{-5}$ |  | 0.345 |  | 1.472 |  |
| Jitter |  | 0.053 |  | 0.010 |  | 0.176 |  | 5125.567 |  | 0.853 |  |
| Memory Load + Jitter |  | 0.053 |  | 0.004 |  | 0.071 |  | 2076.643 |  | 4.895 |  |
| Memory Load + Jitter + Memory Load  ✻  Jitter |  | 0.053 |  | 1.246$e^{-4}$ |  | 0.002 |  | 65.930 |  | 1.626 |  |
| Age group |  | 0.053 |  | 1.056$e^{-4}$ |  | 0.002 |  | 55.877 |  | 1.051 |  |
| Memory Load + Age group |  | 0.053 |  | 3.887$e^{-5}$ |  | 6.997$e^{-4}$ |  | 20.560 |  | 3.097 |  |
| Jitter + Age group |  | 0.053 |  | 0.568 |  | 23.663 |  | 300415.904 |  | 2.423 |  |
| Memory Load + Jitter + Age group |  | 0.053 |  | 0.225 |  | 5.234 |  | 119158.087 |  | 4.232 |  |
| Memory Load + Jitter + Memory Load  ✻  Jitter + Age group |  | 0.053 |  | 0.008 |  | 0.142 |  | 4133.737 |  | 8.833 |  |
| Memory Load + Age group + Memory Load  ✻  Age group |  | 0.053 |  | 1.139$e^{-5}$ |  | 2.049$e^{-4}$ |  | 6.022 |  | 3.690 |  |
| Memory Load + Jitter + Age group + Memory Load  ✻  Age group |  | 0.053 |  | 0.069 |  | 1.343 |  | 36734.367 |  | 2.381 |  |
| Memory Load + Jitter + Memory Load  ✻  Jitter + Age group + Memory Load  ✻  Age group |  | 0.053 |  | 0.002 |  | 0.043 |  | 1275.103 |  | 4.246 |  |
| Jitter + Age group + Jitter  ✻  Age group |  | 0.053 |  | 0.073 |  | 1.412 |  | 38481.087 |  | 2.407 |  |
| Memory Load + Jitter + Age group + Jitter  ✻  Age group |  | 0.053 |  | 0.028 |  | 0.521 |  | 14872.401 |  | 2.096 |  |
| Memory Load + Jitter + Memory Load  ✻  Jitter + Age group + Jitter  ✻  Age group |  | 0.053 |  | 9.849$e^{-4}$ |  | 0.018 |  | 520.958 |  | 3.090 |  |
| Memory Load + Jitter + Age group + Memory Load  ✻  Age group + Jitter  ✻  Age group |  | 0.053 |  | 0.011 |  | 0.200 |  | 5825.142 |  | 13.411 |  |
| Memory Load + Jitter + Memory Load  ✻  Jitter + Age group + Memory Load  ✻  Age group + Jitter  ✻  Age group |  | 0.053 |  | 2.869$e^{-4}$ |  | 0.005 |  | 151.757 |  | 1.929 |  |
| Memory Load + Jitter + Memory Load  ✻  Jitter + Age group + Memory Load  ✻  Age group + Jitter  ✻  Age group + Memory Load  ✻  Jitter  ✻  Age group |  | 0.053 |  | 3.033$e^{-5}$ |  | 5.460$e^{-4}$ |  | 16.044 |  | 2.961 |  |
|  | | | | | | | | | | | |
| *Note.*  All models include subject. | | | | | | | | | | | |
