# Supplementary material for: Older adults preserve accuracy but not precision in explicit and implicit rhythmic timing
Source: PLoS One. 2020 Oct 19;15(10):e0240863. doi: 10.1371/journal.pone.0240863 (PMC7571673; doi:10.1371/journal.pone.0240863)
Supplement: S1 Table — The Bayesian Independent Samples T-test analysis was performed using JASP with the threshold (V2) and age as the dependent and grouping variables. (PDF) [file pone.0240863.s003.pdf]

**S1 Table. Results for the explicit task (Study 1).** The Bayesian Independent Samples T-test analysis was performed using JASP with the threshold (V2) and age as the dependent and grouping variables.

**Bayesian Independent Samples T-Test**

| | $BF_{+0}$ | error % |
|---|---|---|
| Threshold | 4.381 | ~ 2.531e -4 |

*Note.* For all tests, the alternative hypothesis specifies that group *Old* is greater than group *Young* .
