# Supplementary material for: Older adults preserve accuracy but not precision in explicit and implicit rhythmic timing
Source: PLoS One. 2020 Oct 19;15(10):e0240863. doi: 10.1371/journal.pone.0240863 (PMC7571673; doi:10.1371/journal.pone.0240863)
Supplement: S2 Table — Bayesian repeated-measures ANOVA was performed using JASP. The variables age group and jitter were considered to be a between subject factor and repeated measures factor, respectively. (PDF) [file pone.0240863.s004.pdf]

S2 Table. Model comparison results for each participant's response time for the Implicit task (Study 1). Bayesian repeated-measures ANOVA was performed using JASP. The variables age group and jitter were considered to be a between subject factor and repeated measures factor, respectively.

Model Comparison

Models	P(M)	P(M|data)	BF_M	BF_{10}	error %
Null model (incl. subject)	0.200	$9.137e^{-15}$	$3.655e^{-14}$	1.000	
Jitter	0.200	0.655	7.597	$7.170e^{13}$	0.540
Age group	0.200	$3.896e^{-14}$	$1.558e^{-14}$	0.426	0.659
Jitter + Age group	0.200	0.322	1.903	$3.528e^{13}$	3.426
Jitter + Age group + Jitter ✻ Age group	0.200	0.023	0.092	$2.469e^{12}$	6.115

Note. All models include subject.
