# Supplementary material for: Older adults preserve accuracy but not precision in explicit and implicit rhythmic timing
Source: PLoS One. 2020 Oct 19;15(10):e0240863. doi: 10.1371/journal.pone.0240863 (PMC7571673; doi:10.1371/journal.pone.0240863)
Supplement: S3 Table — Bayesian repeated-measures ANOVA was performed using JASP. The variables age group and jitter were considered to be a between subject factor and repeated measures factor, respectively. (PDF) [file pone.0240863.s005.pdf]

S3 Table. Model comparison results for each participant's sensitivity measures (d-prime) for the Implicit task (Study 1). Bayesian repeated-measures ANOVA was performed using JASP. The variables age group and jitter were considered to be a between subject factor and repeated measures factor, respectively.

Model Comparison

Models	P(M)	P(M|data)	BF_M	BF_{10}	error %
Null model (incl. subject)	0.200	$1.203e^{-5}$	$4.813e^{-5}$	1.000	
Age group	0.200	$1.275e^{-5}$	$5.101e^{-5}$	1.060	1.607
Jitter	0.200	0.395	2.607	32794.961	0.542
Age group + Jitter	0.200	0.479	3.682	39837.005	1.362
Age group + Jitter + Age group ∗ Jitter	0.200	0.126	0.577	10475.853	3.070

Note. All models include subject.
