# Supplementary material for: Older adults preserve accuracy but not precision in explicit and implicit rhythmic timing
Source: PLoS One. 2020 Oct 19;15(10):e0240863. doi: 10.1371/journal.pone.0240863 (PMC7571673; doi:10.1371/journal.pone.0240863)
Supplement: S4 Table — Bayesian repeated-measures ANOVA was performed using JASP. While the age group was considered to be a between subject factor, memory load and jitter conditions were specified as factors of repeated measures. (PDF) [file pone.0240863.s006.pdf]

S4 Table. Model comparison results for each participant's response times in the Implicit task with working memory conditions (Study 2). Bayesian repeated-measures ANOVA was performed using JASP. While the age group was considered to be a between subject factor, memory load and jitter conditions were specified as factors of repeated measures.

Model Comparison

Models	P(M)	P(M|data)	BF_M	BF_{10}	error %
Null model (incl. subject)	0.053	$6.569e^{-5}$	0.001	1.000	
Memory Load	0.053	$1.033e^{-5}$	$1.859e^{-4}$	0.157	3.678
Jitter	0.053	0.043	0.807	652.826	0.804
Memory Load + Jitter	0.053	0.007	0.128	107.344	5.892
Memory Load + Jitter + Memory Load ✻ Jitter	0.053	$4.245e^{-4}$	0.008	6.462	2.026
Age group	0.053	$7.291e^{-4}$	0.013	11.100	15.798
Memory Load + Age group	0.053	$9.719e^{-5}$	0.002	1.479	2.338
Jitter + Age group	0.053	0.402	12.111	6122.757	1.473
Memory Load + Jitter + Age group	0.053	0.064	1.223	968.145	5.967
Memory Load + Jitter + Memory Load ✻ Jitter + Age group	0.053	0.004	0.080	67.426	6.706
Memory Load + Age group + Memory Load ✻ Age group	0.053	$2.388e^{-5}$	$4.298e^{-4}$	0.363	1.716
Memory Load + Jitter + Age group + Memory Load ✻ Age group	0.053	0.018	0.329	273.222	4.746
Memory Load + Jitter + Memory Load ✻ Jitter + Age group + Memory Load ✻ Age group	0.053	0.001	0.021	17.400	2.869
Jitter + Age group + Jitter ✻ Age group	0.053	0.381	11.095	5804.935	1.177
Memory Load + Jitter + Age group + Jitter ✻ Age group	0.053	0.057	1.083	863.955	1.895
Memory Load + Jitter + Memory Load ✻ Jitter + Age group + Jitter ✻ Age group	0.053	0.004	0.070	58.936	1.829
Memory Load + Jitter + Age group + Memory Load ✻ Age group + Jitter ✻ Age group	0.053	0.016	0.296	246.009	3.180
Memory Load + Jitter + Memory Load ✻ Jitter + Age group + Memory Load ✻ Age group + Jitter ✻ Age group	0.053	0.001	0.020	16.778	2.294
Memory Load + Jitter + Memory Load ✻ Jitter + Age group + Memory Load ✻ Age group + Jitter ✻ Age group + Memory Load ✻ Jitter ✻ Age group	0.053	$1.710e^{-4}$	0.003	2.603	2.101

Note. All models include subject.
