# Supplementary material for: Older adults preserve accuracy but not precision in explicit and implicit rhythmic timing
Source: PLoS One. 2020 Oct 19;15(10):e0240863. doi: 10.1371/journal.pone.0240863 (PMC7571673; doi:10.1371/journal.pone.0240863)
Supplement: S5 Table — Bayesian repeated-measures ANOVA was performed using JASP. While the age group was considered to be a between subject factor, memory load and jitter conditions were specified as factors of repeated measures. (PDF) [file pone.0240863.s007.pdf]

S5 Table. Model comparison results for each participant's sensitivity measures (d-prime) in the Implicit task with working memory conditions (Study 2). Bayesian repeated-measures ANOVA was performed using JASP. While the age group was considered to be a between subject factor, memory load and jitter conditions were specified as factors of repeated measures.

Model Comparison

Models	P(M)	P(M|data)	BF_M	BF_{10}	error %
Null model (incl. subject)	0.053	$1.891e^{-6}$	$3.4032e^{-5}$	1.000	
Memory Load	0.053	$6.528e^{-7}$	$1.175e^{-5}$	0.345	1.472
Jitter	0.053	0.010	0.176	5125.567	0.853
Memory Load + Jitter	0.053	0.004	0.071	2076.643	4.895
Memory Load + Jitter + Memory Load ✻ Jitter	0.053	$1.246e^{-4}$	0.002	65.930	1.626
Age group	0.053	$1.056e^{-4}$	0.002	55.877	1.051
Memory Load + Age group	0.053	$3.887e^{-5}$	$6.997e^{-4}$	20.560	3.097
Jitter + Age group	0.053	0.568	23.663	300415.904	2.423
Memory Load + Jitter + Age group	0.053	0.225	5.234	119158.087	4.232
Memory Load + Jitter + Memory Load ✻ Jitter + Age group	0.053	0.008	0.142	4133.737	8.833
Memory Load + Age group + Memory Load ✻ Age group	0.053	$1.139e^{-5}$	$2.049e^{-4}$	6.022	3.690
Memory Load + Jitter + Age group + Memory Load ✻ Age group	0.053	0.069	1.343	36734.367	2.381
Memory Load + Jitter + Memory Load ✻ Jitter + Age group + Memory Load ✻ Age group	0.053	0.002	0.043	1275.103	4.246
Jitter + Age group + Jitter ✻ Age group	0.053	0.073	1.412	38481.087	2.407
Memory Load + Jitter + Age group + Jitter ✻ Age group	0.053	0.028	0.521	14872.401	2.096
Memory Load + Jitter + Memory Load ✻ Jitter + Age group + Jitter ✻ Age group	0.053	$9.849e^{-4}$	0.018	520.958	3.090
Memory Load + Jitter + Age group + Memory Load ✻ Age group + Jitter ✻ Age group	0.053	0.011	0.200	5825.142	13.411
Memory Load + Jitter + Memory Load ✻ Jitter + Age group + Memory Load ✻ Age group + Jitter ✻ Age group	0.053	$2.869e^{-4}$	0.005	151.757	1.929
Memory Load + Jitter + Memory Load ✻ Jitter + Age group + Memory Load ✻ Age group + Jitter ✻ Age group + Memory Load ✻ Jitter ✻ Age group	0.053	$3.033e^{-5}$	$5.460e^{-4}$	16.044	2.961

Note. All models include subject.
